# The Impact of CKD on Uremic Toxins and Gut Microbiota

**DOI:** 10.3390/toxins13040252

**Published:** 2021-03-31

**Authors:** Jacek Rysz, Beata Franczyk, Janusz Ławiński, Robert Olszewski, Aleksanda Ciałkowska-Rysz, Anna Gluba-Brzózka

**Affiliations:** 1 Department of Nephrology, Hypertension and Family Medicine, Medical University of Lodz, 90-549 Lodz, Poland; jacek.rysz@umed.lodz.pl (J.R.); bfranczyk-skora@wp.pl (B.F.); 2 Department of Urology, Institute of Medical Sciences, Medical College of Rzeszow University, 35-055 Rzeszow, Poland; janlaw@wp.pl; 3 Department of Gerontology, Public Health and Didactics, Rheumatology and Rehabilitation, National Institute of Geriatrics, 02-637 Warsaw, Poland; robert.olszewski@spartanska.pl; 4 Department of Ultrasound, Institute of Fundamental Technological Research, Polish Academy of Sciences, 02-637 Warsaw, Poland; 5 Palliative Medicine Unit, Department of Oncology, Medical University of Lodz, 90-549 Lodz, Poland; olarysz@rmed.pl

**Keywords:** chronic kidney disease, uremic toxins, gut microbiota, cardiovascular risk

## Abstract

Numerous studies have indicated that the progression of chronic kidney disease (CKD) to end-stage renal disease (ESRD) is strictly associated with the accumulation of toxic metabolites in blood and other metabolic compartments. This accumulation was suggested to be related to enhanced generation of toxins from the dysbiotic microbiome accompanied by their reduced elimination by impaired kidneys. Intestinal microbiota play a key role in the accumulation of uremic toxins due to the fact that numerous uremic solutes are generated in the process of protein fermentation by colonic microbiota. Some disease states, including CKD, are associated with the presence of dysbiosis, which can be defined as an “imbalanced intestinal microbial community with quantitative and qualitative changes in the composition and metabolic activities of the gut microbiota”. The results of studies have confirmed the altered composition and functions of gut microbial community in chronic kidney disease. In the course of CKD protein-bound uremic toxins, including indoxyl sulfate, p-cresyl glucuronide, p-cresyl sulfate and indole-3-acetic acid are progressively accumulated. The presence of chronic kidney disease may be accompanied by the development of intestinal inflammation and epithelial barrier impairment leading to hastened systemic translocation of bacterial-derived uremic toxins and consequent oxidative stress injury to the kidney, cardiovascular and endocrine systems. These findings offer new therapeutic possibilities for the management of uremia, inflammation and kidney disease progression and the prevention of adverse outcomes in CKD patients. It seems that dietary interventions comprising prebiotics, probiotics, and synbiotics could pose a promising strategy in the management of uremic toxins in CKD.

## 1. Introduction

Some disease states, including chronic kidney failure, chronic kidney disease (CKD), obesity, diabetes, cardiovascular disease, inflammatory bowel disease, and cancers are associated with the presence of dysbiosis, which can be defined as an “imbalanced intestinal microbial community with quantitative and qualitative changes in the composition and metabolic activities of the gut microbiota” [1,2,3,4]. The results of studies confirm that the composition and functions of gut microbial community are altered in chronic kidney disease [5,6,7]. Numerous studies have indicated that the progression of CKD to end-stage renal disease (ESRD) is strictly associated with the accumulation of toxic metabolites in blood and other metabolic compartments as a result of their enhanced generation from the dysbiotic microbiome accompanied by their reduced elimination by impaired kidneys [1,8,9,10], however, this is not the primary cause of the progression to ESRD. Some uremic solutes were demonstrated to be generated in the process of protein fermentation by colonic microbiota [11].

The intestinal dysbiosis resulting from the loss of kidney function is associated with the secretion of urea into the gastrointestinal tract and successive hydrolysis of urea by some gut microbes [2]. Consequent generation of large amounts of ammonia (and formation of its by-product) rises intestinal pH resulting in mucosal irritation and could have a negative impact on the growth of commensal bacteria favoring the maintenance of intestinal dysbiosis [12,13]. Due to its toxicity, ammonia is converted to urea through the ornithine-urea cycle. Gut bacteria can cleave urea into carbon dioxide and ammonia which is further partly used for microbial synthesis of amino acids and the rest enters host circulation to serve as substrate for synthetic processes [14,15]. In patients with advanced stages of CKD only minimal rise in serum levels of urate is observed due to the presence of CKD-stimulated adaptive secretion of uric acid by the colon [16]. In consequence, in ESRD patients the abundance of bacteria having urease, uricase, and indole and p-cresol forming enzymes, is observed [6]. Moreover, higher concentrations of ammonia destroys the intestinal epithelial tight junction and impairs its barrier function increasing intestinal permeability [17,18,19]. The worsening of intestinal barrier dysfunction in ESRD patients or those undergoing peritoneal dialysis may also be the result of frequent occurrence of edema and hypervolemia [20]. It has been demonstrated that excessive ultrafiltration volumes and/or intradialytic hypotension causing episodes of transient intestinal ischemia could aggravate the dysfunction and permeability of the gut barrier in ESRD patients on dialysis [21,22]. Impairment of colonic epithelial tight junction may enable the aforementioned translocation of bacteria and endotoxin through the intestinal wall into the underlying tissue compartments, which could activate innate immunity and trigger a local inflammatory process contributing to perpetuating gut barrier damage [23,24]. Indeed, the results of animal studies have confirmed significant depletion of the vital protein constituents of the epithelial tight junction, enhanced systemic oxidative stress, and the penetration of bacteria across the intestinal wall [25].

CKD-related alterations of gut microbial community were also suggested to be related to dietary restrictions aiming at the prevention of hyperkalemia as well as and malnutrition. Moreover, patients with CKD are frequently polymedicated [20]. The frequent use of antibiotics was shown to modify the relative count of bacterial community members reducing their diversity [26]. Some drugs prescribed to this group of patients may alter intestinal microflora, while others, such as phosphorus binders and ion exchange resins may additionally slow intestinal transit [27].

However, the question of the exact mechanisms related to gut microbiota-mediated changes in ESRD metabolome remains unanswered [10]. The unravelling of these mechanisms may have therapeutic significance since according to some researchers the change of specific gut microbes may translate into the regulation of circulating uremic toxin concentrations [28].

## 2. Gut Microbiome in Health

Over 100 trillion microbial cells are present in the human gut constituting the gut microbiota [2]. The results of the Metagenomics of the Human Intestinal Tract (Meta-HIT) project and Human Microbiome Project (HMP) indicate that the human intestine is inhabited by an extremely complex and dynamic consortium of bacteria exerting vital impact on human health and disease states [1,29,30]. It is estimated that average individual’s unique gut microbiota comprises 500 to 1000 bacterial species [31]. Every species of bacteria colonizes a particular niche and therefore the composition of bacteria along the intestinal tract is diverse. Bacteroidetes, Actinobacteria and Firmicutes were shown to be the predominant bacterial groups present in the human gastrointestinal tract [32,33].

The intestinal epithelium, which is a single layer of columnar epithelial cells, plays a vital role in nutrient absorption, as well as acting as a natural barrier preventing or hampering systemic translocation of pathogens and antigens [34]. Intestinal epithelial barrier function is enhanced by probiotic bacteria, while commensal bacteria preserve this barrier via suppressing intestinal inflammation [35,36].

Gut microbiota is involved in multiple functions, for example the synthesis of certain vitamins (K and B groups), the breakdown of indigestible plant polysaccharides, the activation of bioactive food components (flavonoids, isoflavanoids, and plant lignans), the degradation of dietary oxalates and the biotransformation of conjugated bile acids, which contributes to the nutritional balance [37,38,39,40,41]. Commensal gut microbes preserve the functional integrity of the gut via the restoration of tight junction protein structure, the upregulation of mucin genes, the stimulation of epithelial heat-shock proteins as well as the competition with pathogenic bacteria for the binding to intestinal epithelial cells and the secretion of bacteriocins providing resistance or resilience to infection by pathogens [2,42,43,44,45]. Moreover, the commensal bacteria by suppressing intestinal inflammation and stress-induced damage via the toll-like receptors (TLRs)-mediated pathway, contribute to the maintenance of intestinal epithelial barrier integrity and epithelial homeostasis [36,46]. Short chain fatty acids (SCFA) (such as acetate, propionate, and butyrate), which are the products of anaerobic bacterial fermentation of dietary polysaccharides, enter systemic circulation via passive diffusion in colonocytes and active transport mechanisms, where they exert impact on immune system regulation, energy metabolism, and blood pressure [47]. Some researchers have found that the treatment of mice (model of acute kidney injury) with SCFA limits ischemia reperfusion injury (IRI) via the reduction of inflammation diminishing in consequence kidney injury [48,49]. The results of recent studies indicate that gut microbiota can even modulate the functionality of the central nervous system [50].

## 3. Gut Microbiome in CKD

In the course of CKD, microbiome composition and the intestinal environment undergo transformation from a symbiotic to a dysbiotic state which is mirrored by an increase in colonic protein fermentation and resulting rise in microbiota-derived uremic toxins as well as a decrease in carbohydrate fermentation and subsequent diminished formation of host-beneficial metabolites (e.g., SCFAs) [5,6]. According to studies, in uremic patients excessive colonization of the duodenum and jejunum by much higher counts of aerobic (approximately 106 bacteria/mL) and anaerobic (approximately 107 bacteria/mL) organisms is observed in CKD patients compared to healthy persons [51]. Vaziri et al. [5] demonstrated significantly higher abundance of 190 bacterial operational taxonomic units (OTUs) from Brachybacterium, Enterobacteriaceae, Catenibacterium, Moraxellaceae, Polyangiaceae, Halomonadaceae, Thiothrix, Nesterenkonia and Pseudomonadaceae families in end-stage renal disease compared to apparently healthy controls. The results of other studies showed an increased number of aerobic bacteria, such as Enterobacteria and Enterococci species and considerably lower number of Bifidobacterium species in patients undergoing maintenance hemodialysis compared to controls [7]. Hida et al. [7] observed significantly higher amount of Clostridium perfringens in these patients. According to Wong et al. [6], out of 19 microbial families which were found to be dominant in ESRD patients, 12 possessed urease (Alteromonadaceae, Clostridiaceae, Cellulomonadaceae, Dermabacteraceae, Halomonadaceae, Enterobacteriaceae, Methylococcaceae, Moraxellaceae, Micrococcaceae, Polyangiaceae, Xanthomonadaceae, and Pseudomonadaceae), five had uricase (Cellulomonadaceae, Micrococcaceae, Dermabacteraceaea, Xanthomonadaceae and Polyangiaceae families), while there had indole and p-cresyl-forming enzymes (i.e., tryptophanase possessing families: Clostridiaceae, Verrucomicrobiaceae, and Enterobacteriaceae). In turn, Wang et al. [10] indicated that Eggerthella lenta, which degrade polyphenols into precursors of hippuric acid: benzoic acid or 4-hydroxybenzoic acid, as well as Fusobacterium nucleatum participating in the formation of indole and phenol, were the most enriched species in patients with ESRD [10,52]. Moreover, also Alistipes shahii, an indole-positive bacterium, and Clostridium difficile (chief producer of p-cresol) were also identified among ESRD-enriched bacteria [53,54]. However, some studies failed to observe significant differences in total counts of microorganisms, but, they demonstrated the displacement of the aerobic bacteria by the anaerobic bacteria (especially Lactobacillus and Bifidobacterium) and subsequent stimulation of degradation of nitrogen compounds in deteriorative uremic state [18,55]. In turn, Joossens et al. [56] found that in ESRD the composition of the gut microbiota is not uniform but it lacks characteristic microbial signature.

The presence of dysbiotic gut microbiome is not only associated with enhanced production of harmful metabolites and uremic toxins, but also with the cessation of beneficial metabolites (e.g., SCFA) formation. Wong et al. [6] reported that two families (Prevotellaceae and Lactobacillaceae) having SCFA (butyrate) forming enzymes, were amongst the four microbial families depleted in ESRD patients [6].

**Figure 1:**
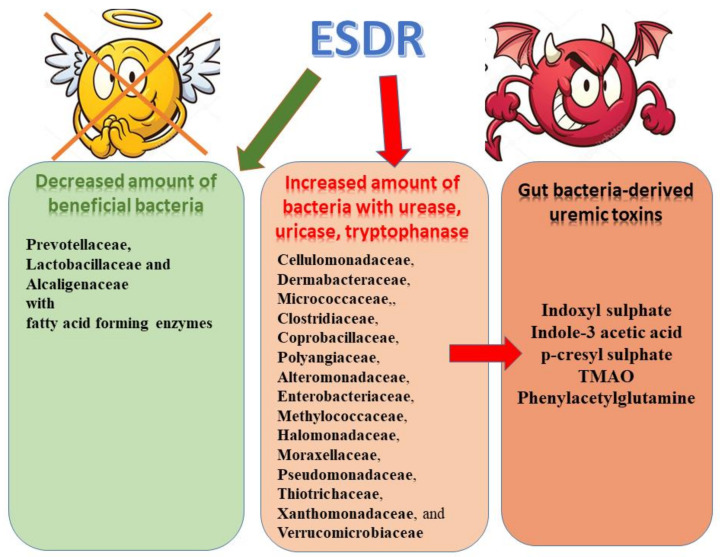
Microbiome alterations observed in ESRD patients and uremic toxins.

Therefore, it seems that in general, CKD-related alterations upregulate microbiota producing specific uremic toxins and downregulate microbiota generating beneficial products [50]. This thesis was confirmed by Poesen et al. [57] who observed higher generation of indoles, p-cresol, benzenes, aldehydes, furans, and branched-chain, medium-chain, and short-chain fatty acids and a reduced generation of ketones in fecal metabolite profiles of patients on hemodialysis. However, he suggested that alteration of colonic microbial metabolism observed in CKD was associated with diet, and to a lesser extent with the loss of kidney function [57]. In turn, Gryp et al. [58] implied that the enhanced accumulation of uremic toxins in plasma of CKD patients was not the result of bacterial generation of indole, p-cresol, and IAA. According to them some patients may generate larger amounts of specific protein-bound uremic toxins precursors than others, irrespective of kidney function [58]. However, the results of study carried out by Wang et al. [10] provide evidence that greatly altered gut microbiota in patients with renal failure could be associated with accelerated biosynthesis of numerous toxic compounds, and consequent increase in plasma concentrations of uraemic toxins and intensified kidney disease. Figure 1 presents the changes in bacterial counts in ESRD.

## 4. Adverse Effects of Gut-Derived Uremic Toxins and Microbial Metabolites

The development of gut microbiome dysbiosis in CKD patients could result in impaired protein assimilation, prolonged intestinal transit time, intestinal wall edema, metabolic acidosis, iron therapy and diminished consumption of dietary fiber [27,59,60,61]. According to studies, impaired protein assimilation in uremia may contribute to malnutrition and is associated with enhanced influx of undigested proteins into the distal intestine promoting the proliferation of proteolytic bacteria and microbial fermentation [59,62,63]. The consequences of amplified protein fermentation involve the formation of potentially toxic metabolites, including indoles, phenols, ammonia, thiols, and amines. Increased intestinal barrier permeability facilitates the penetration of uraemic toxin precursors into the circulation resulting in the aggravated accumulation of serum toxins and consequent aggravation of kidney disease [64,65]. Uremic toxins exert detrimental effects on various cell types, including kidney tubular cells, immunological cells, endothelial cells as well as bone cells [66,67,68,69,70]. Their accumulation is associated with the protein-energy wasting, the progression of CKD, cardiovascular disease, inflammation, neurologic disorders, vascular calcification, increased mortality from cardiovascular disease and overall mortality [9,50,71,72,73,74,75,76,77,78]. An extensive literature search revealed that, so far, at least 150 different uremic retention solutes have been discovered [79]. Uremic toxins can be categorized on the basis of their physicochemical characteristics into low water-soluble molecules (molecular weight < 500 Da), larger middle molecules (molecular weight > 500 Da), and protein-bound molecules, while based on their site of origin into: endogenous (mammalian metabolism), exogenous (diet) or microbial. The group of main gut-derived uremic toxins comprises indoxyl sulphate (IS) with molecular weight of 213 Da, p-cresyl sulphate (PCS) with molecular weight of 188 Da, indole-3 acetic acid (IAA), trimethylamine N-oxide (TMAO), and phenylacetylglutamine with molecular weight of 264.27 Da [4]. In the course of chronic kidney disease protein-bound uremic toxins (PBUT), including indoxyl sulfate, p-cresyl glucuronide (pCG), p-cresyl sulfate and indole-3-acetic acid are progressively accumulated [58,79,80]. They are generated as products of intestinal microbial metabolism of the aromatic amino acids–tryptophan, tyrosine and phenylalanine [11,81,82]. Tyrosine and phenylalanine are metabolized in the distal part of the colon into p-cresol, while tryptophan is metabolized by intestinal bacteria such as Escherichia coli into indole and IAA and after intestinal absorption it is further converted in the liver to indoxyl sulphate which is normally excreted in urine [83]. In the colon mucosa and liver, p-cresol and indole undergo partial detoxification via sulfation by host, and pCS and IS are formed. A small portion of p-cresol is also detoxificated via glucuronidation, which results in the creation of pCG [81,84]. Indoles are the group of aromatic compounds comprising a pyrrole ring [1]. In the gut, more than 600 indoles have been found. In healthy persons, indoxyl sulfate (IS) (which belongs to aforementioned group) is removed by proximal tubules of the kidneys; however, in patients with renal impairment it becomes accumulated probably as the result of reduced cellular transport mediated by organic anion transporters OAT1 and OAT3, which may lead to further tubular damage and chronic kidney disease progression [67,73,85,86,87,88]. In healthy kidneys, glomerulus participate in the filtration of blood and retaining essential plasma proteins ensuring selective ultrafiltration [89]. The glomerular filter contains three important layers the fenestrated endothelium, the intervening glomerular basement membrane and the epithelial podocytes [90]. The glomerular basement membrane (GBM) offers the primary structural support for the glomerular tuft containing glomerular capillary. Endothelial and smooth muscle-like mesangial cells are located inside the GBM, while podocytes are attached on the outer surface of the GBM [89,91]. Such structure enables the flow of plasma water and small molecules, and form a size selectivity barrier for the passage of larger molecules [92]. Renal diseases are associated with impairment of this structure and its greater permeability for larger molecules.

In normal conditions, uremic toxins which bind with plasma albumin are excreted into urine via by organic anion and cation transporters present in the kidney tubular cells (tubular secretion) which seems to be the predominating mechanism [93,94]. In turn, unbound toxins are removed through glomerular filtration. However, in patients with CKD such elimination of toxins is reduced due to the renal impairment which results in their blood accumulation [73,85,95]. They usually cannot be efficiently removed by dialysis. Indeed, the reduction rate of IS and PCS are 31.8 and 29.1%, respectively, with regular hemodialysis treatment [96]. Toxins, urea and other small particles can pass through an artificial filter containing semi-permeable dialysis membrane. The transfer of metabolic toxins through the membrane into the dialysis fluid is based on diffusion, i.e., moving of molecules contained in blood and dialysis fluid through a semi-permeable membrane to the solution with lower concentration. The efficiency of the removal of uremic toxins, especially middle molecular weight molecules, has been upgraded with the introduction of a high-flux dialyzer membrane during hemodialysis [97,98].

Reduced fractional kidney clearance implies that tubular clearance is more disturbed than glomerular filtration. Therefore, it is not surprising that the highest levels of uremic toxins are found in end-stage renal disease undergoing dialysis [85].

Indole is an intercellular signal molecule which modulates diverse biological functions; for example it upregulates gene expression of gut epithelial cell junctions and controls expression of pro- and anti-inflammatory factors in intestinal epithelial cells therefore enabling the maintenance of host-microbe homeostasis at the mucosal surface [58,99,100,101]. In patients with CKD levels of indole acetic acid (IAA) are also elevated due to the fact that this protein-bound uremic solute is only partly removed during hemodialysis [102]. IAA generated from tryptophan by intestinal bacteria and normal cells was found to stimulate glomerular sclerosis and interstitial fibrosis leading to the progression of CKD [66]. It induces proinflammatory enzyme c‘yclooxygenase-2 and oxidative stress and its concentration enables the prediction of mortality and cardiovascular events in patients with CKD [77]. In renal proximal tubular cells, IS stimulates the expression of nuclear factor (NF)-κB and plasminogen activator inhibitor type 1 which results in nephrotoxic effects as well as enhancing the expression of tissue inhibitor of metalloproteinases and transforming growth factor (TGF)β1 involved in tubulointerstitial fibrosis [88,103,104]. Furthermore, increased levels of IS have been suggested to be associated with enhanced oxidative stress in endothelial cells, vascular smooth muscle cell proliferation, vascular stiffness, aortic calcification and overall and cardiovascular mortality in patients with CKD [9,105,106]. In hemodialysis patients, IS and PCS increases the risk of peripheral vascular disease and thrombosis of vascular access [107]. Moreover, IS was demonstrated to exert cardiac profibrotic effect, to favor myocardiocytes hypertrophy and to predispose to atrial fibrillation [20,108,109]. Adverse effects of IS also include also inhibitory effect on osteoclast function, the development of low-turnover bone disease, and impact on bone formation rate [110,111]. IS-related decrease in bone formation results from the stimulation of oxidative stress in osteoblasts and the induction of resistance to parathormon (PTH), which favors the development of adynamic bone [112]. Lin et al. [113] observed a positive correlation between fibroblast growth factor 23 (FGF-23) and IS serum levels, which may imply the link between this molecule and metabolic bone disease in uraemic patients. Finally, the relationship between IS and CKD-related anemia has been observed since it diminishes erythropoiesis, hampers the activity of erythropoietin and enhances programmed cell death of red blood cells (eryptosis) [114,115,116].

Phenols are other aromatic compounds which are produced by intestinal bacteria (Lactobacillus, Bacterioides, Enterobacter, Bifidobacterium and Clostridium) via partial breakdown of tyrosine and phenylalanine [117]. The urinary excretion of p-cresol sulfate (PCS) depending on tubular secretion through specific transporters is impaired in CKD patients resulting in the accumulation of this uremic toxin [118,119,120]. P-cresol is a potent inhibitor of biological functions [68,121]. Together with the IS, they have been demonstrated to activate intrarenal renin-angiotensin system and TGF/Smad pathway promoting kidney fibrosis [122]. In turn, the results of animal studies indicated that PCS was associated with severe tubular damage in 5/6-nephrectomized rats since it enhanced oxidative stress and inflammatory cytokines levels [123]. Increased levels of this uremic toxin increases the risk of all-cause mortality and cardiovascular disease in patients with chronic kidney failure and mild-to-moderate CKD [76,124]. Also, Lin et al. [78] also demonstrated negative relationship between levels of indoxyl sulfate and p-cresyl sulfate in the serum and kidney function. Both toxins, p-cresyl sulfate and indoxyl sulfate have been shown to exert multimodal detrimental effects, including the stimulation of oxidative stress, fibrosis, and inflammatory responses [125,126,127]. High plasma levels of PCS and IS are correlated with the progression to end-stage renal disease and enhanced mortality in CKD patients [9,81,128]. A longitudinal study demonstrated strong correlation between variations in peripheral levels of p-cresyl conjugates (the composite of p-cresyl sulfate (pCS)/glucuronide (pCG)), indoxyl sulfate (IS), indole acetic acid and creatinine and fecal microbial community divergence [56]. Levels of the two foremost uraemic toxins, IS and pCS were found to be associated with the overall bacterial community composition (*p* < 0.05) [56].

Rossi et al. [129] reported the relationship between indoxyl sulphate and p-cresyl sulphate and increased inflammatory biomarkers in stage 3–4 CKD patients, such as glutathione peroxidase and interleukin-6. Moreover, the pretreatment of human umbilical vein endothelial cells (HUVECs) with IS pretreatment considerably increased interleukin IL-1β-induced E-selectin expression, monocyte adhesion, and the phosphorylation of mitogen-activated protein kinases (ERK, p38, and JNK) and transcription factors (NF-κB and AP-1) [130]. Besides, IS enhanced IL-1β-induced reactive oxygen species (ROS) production and this effect was inhibited by pretreatment with a ROS scavenger (N-acetylcysteine) or a NADPH oxidase inhibitor (apocynin). Experimental data from animal studies indicate that IS and PCS stimulate renal fibrosis and are responsible for severe renal tubular damage in CKD rats [126]. In turn, toxins produced by E. lenta and F. nucleatum were associated with the worsening of renal fibrosis in 5/6 nephrectomy rat models. A prospective study of patients with stage 1–5 CKD confirmed the predictive role of IS and PCS in CKD progression [73].

Apart from the aforementioned, also other toxins (trimethylamine-N-oxide, hippuric acid, hydrogen sulfide, phenylacetylglutamine and phenyl sulfate) also come from bacterial metabolism [58,82,131]. Phenylacetylglutamine (PAG) is a colonic microbe-derived nitrogenous metabolite, received in the course of phenylalanine fermentation. Its accumulation is observed in uremic patients [132]. This compound is generated in the liver via the metabolism of toxic phenylacetic acid, derived from phenylalanine. In turn, the administration of phenyl sulfate has been shown to induce albuminuria and podocyte damage in experimental models of diabetes [133]. Moreover, in diabetic patient phenyl sulfate concentration considerably correlated with basal and predicted two-year progression of albuminuria in patients with microalbuminuria. Following the inhibition of bacterial enzyme responsible for the synthesis of phenol from dietary tyrosine (tyrosine phenol-lyase) reduced albuminuria in diabetic mice was observed [133].

Finally, TMAO (molecular weight 75 kDa) is a gut-derived toxic metabolite obtained as a product of bacterial metabolism of quaternary amines (betaine, l-carnitine or phosphatidylcholine). Trimethylamine formed in the first step is later absorbed and converted to TMAO by flavin monooxygenase enzymes in the liver [134]. Trimethylamine-N-Oxide (TMAO) was demonstrated to predict an augmented risk of major adverse cardiovascular events after the adjustment for traditional risk factors, as well as being associated with higher risk of death in CKD patients [135,136]. Blood levels of TMAO positively correlate with the presence of Clostridiaceae and Peptostreptococcaceae families, but also with increased atherosclerosis risk [137]. TMAO has been suggested to alter the metabolism of cholesterol, stimulate the expression of scavenger receptors on macrophages promoting foam cell formation, modify bile acid metabolism and affect sterol transporters in the liver and intestine [137,138]. The results of animal studies revealed that the administration of TMAO resulted in tubulointerstitial fibrosis and collagen deposition [136]. Moreover, increased TMAO levels were found to predict coronary atherosclerotic burden and higher mortality in patients with CKD [136,139,140].

It has been demonstrated that dialysis patients may suffer from the toxin-induced residual uraemic syndrome, severely affecting the quality of their lives, due to the fact that traditional renal replacement therapy does not efficiently remove these toxins [141]. Some authors have suggested that the generation of uremic toxins acts as a precursors to changes in the progression of CKD [142,143]. However, Gryp et al. [58] demonstrated that in CKD patients, absolute levels of aromatic amino acids as well as their metabolites (indole, p-cresol, IAA) did not differ significantly among stages. However, plasma PBUT levels, but not urinary concentrations, increased with advancing stages of CKD which may imply that bacterial generation of p-cresol and indole do not contribute to the elevated plasma PBUT levels. The increase in the generation of intestinal generation of protein-bound uremic toxins precursor metabolites could be associated with higher PBUT concentrations in CKD. The results of studies concerning adverse effects of uremic toxins are summarized in Table 1.

## 5. Therapeutic Potential

The diagnosis as well as the treatment and prevention of some diseases requires the knowledge of the composition, dynamics, and stability of gut microbiota [2]. Numerous studies have suggested that the change of diet could potentially diminish uremic toxin levels [144,145,146,147]. The administration of pre-, pro-, and synbiotics have been shown to exert positive effects on cresol and indole metabolism [148,149,150]. According to the definition of the United Nations’ Food and Agriculture Organization and the World Health Organization probiotics are “live microorganisms” which when administered in adequate amounts bring health benefits to the host [151].

### 5.1. Probiotics

Probiotics contain i.e., living species of Bifidobacteria, lactobacilli, and streptococci which are capable of altering gut microbiota and soothing the inflammatory state [152,153]. The efficacy of probiotics has been confirmed in many studies, but not all. Oral administration of Lactobacillus acidophilus resulted in reduced serum dimethylamine (potential uremic toxin) in hemodialysis patients [51]. The administration of Bifidobacterium longum in the form of enteric capsules was shown to be effective in decreasing the pre-HD serum levels of homocysteine, indoxyl sulfate, and triglyceride, but exerted minimal effects on CKD progression in these patients [154]. The results of randomized, double-blind trial enrolling patients on peritoneal dialysis also demonstrated a significant decrease in serum proinflammatory endotoxin and cytokine levels (TNF-α, IL-5, IL-6), an rise in serum IL-10 levels, as well as the preservation of residual renal function after six months of treatment with a probiotic [155].

### 5.2. Prebiotics

Numerous studies have indicated that also the intake of prebiotics, which are non-digestible food ingredients stimulating the growth or activity of bacteria in the colon, may also significantly decrease the generation of uremic toxins. Prebiotics stimulate the growth of Bifidobacteria and Lactobacilli species at the expense of other groups of bacteria in the gut [156]. The consumption of a mixture of probiotics and prebiotics has been demonstrated to lower plasma levels of PCS and IS and to exert probable renoprotective effects [150,154,157]. For example, oligofructose inulin was shown to greatly diminish the generation of PCS and its serum levels in HD patients [150]. The results of studies demonstrated that a high fiber diet enhances the production of SCFA, which provides energy to the intestinal flora and enables the incorporation of amino acids reaching the colon into bacterial proteins and their successive excretion instead of being fermented into uraemic solutes [20]. Moreover, SCFAs are used as substrate by the intestinal mucosa facilitating the maintenance of their functionality and integrity. Rossi et al. [158] found that a direct relationship between dietary protein/fiber ratio and PCS and IS levels in CKD patients. Also, a pilot prospective, randomized, double-blind, placebo-controlled multinational crossover trial assessing the efficacy of Renadyl formulation containing Lactobacillus acidophilus, Streptococcus thermophilus and Bifidobacterium longum in patients with CKD stages 3 and 4 also revealed that six months therapy was associated with considerably decreased blood urea and improved life quality [159]. However, the follow-up randomized controlled trial failed to reduce plasma uremic toxins and to improve life quality [160]. The benefits of probiotics could be associated with the substitution of persistent uremia-induced alterations in gut biochemical milieu by the symbiotic microbiota [64].

### 5.3. Synbiotics

The use of synbiotics (a mixture of probiotics and prebiotics beneficially affecting the host by ameliorating the survival and activity of beneficial microorganisms in the gut) was proved to be beneficial in CKD. A trial investigating the combination of probiotic and prebiotic therapies for over six weeks in pre-dialysis CKD patients found that such therapy lowered serum p-cresyl sulphate and gut microbiome alterations, but it did not diminish considerably serum IS [161]. The reduction in PCS and IS levels was more marked in patients who did not receive antibiotics during the study. Moreover, synbiotics were also shown to alter the stool microbiome (the enrichment of Bifidobacterium and the depletion of Ruminococcaceae). The choice of probiotic microbe is important in terms of the anticipated effects [64,162]. Four-week treatment with synbiotic Probinul neutron reduced total plasma p-cresol but it did not affect gastrointestinal symptoms in stage 3–4 CKD patients [148]. In turn, a SYNERGY trial revealed a lowering in serum p-cresyl sulphate but not in indoxyl sulphate as well as a positive change in stool microbiome in stage 4–5 CKD patients [161]. In this trial, an increase in Bifidobacteria and decrease of fecal Ruminococcus was observed. However, the treatment did not change the levels of inflammation markers, oxidative stress, or endotoxins, but a slight increase in albuminuria was noticed. The administration of the combination of Lactobacillus casei strain Shirota and Bifidobacterium breve strain Yakult with galacto-oligosaccharides also considerably decreased serum p-cresol and improvement of stools quantity and quality in hemodialysis patients [55]. Mishima et al. [163] reported that the use of lubiprostone (laxative drug) improved renal damage in CKD via the alterations in intestinal environment and decreasing uremic toxin levels which implies that the modulation of intestinal environment could be used as an effective therapy for CKD. In their subsequent study, canagliflozin therapy was shown to diminish plasma levels of uremic toxins (via the inhibition of sodium-dependent glucose cotransporters: SGLT2, but also probably SGLT-1), especially PCS and to alter intestinal metabolites in animal model of CKD [127]. The observed effect could be associated with the fact that canagliflozin increased colonic SCFA levels and changed gut microbial composition. Other studies also indicated canagliflozin-related slowing of renal function decline progression independently of glycemic effects [164,165]. Renoprotective effect of canagliflozin may result from the reduction of PCS levels which translate into diminished risk of end-stage renal disease as well as that of cardiovascular diseases [128,166]. Finally, the use of oral sorbents has been suggested to reduce concentrations of uraemic toxins and circulating intestinal endotoxins. AST-120 (oral activated charcoal adsorbent) was reported to lower IS levels in a dose-dependent manner in Japan [167]. Moreover, a considerable decrease in the IS, PCS, or phenyl sulfate and oxidative stress levels have been revealed in HD patients, but this effect disappeared after the discontinuation of AST-120 [168]. The use of AST-120 also resulted in substantial lowering of the oxidized albumin and 8-isoprostane levels. Therefore, it seems that oral administration of AST-120 exerts additive effects on the continuous decline of some PBUTs levels in anuric HD patients. The addition of AST-120 to continuous erythropoietin receptor activator treatment has been suggested to improve renal function and hemoglobin levels compared to CERA alone in late-stage CKD patients. Beneficial eGFR-related effects could be associated with lower serum IS/PCS and improved hemoglobin levels. Other authors have also demonstrated that the intake of AST-120 ameliorated erythropoietic response to CERA [169]. The results of a retrospective study of the long-term effects of AST-120 confirmed a decreased risk of progression to dialysis, mortality, cardiac and vascular events in patients with stage 3–5 CKD taking AST-120 compared to those who did not receive it [170]. Finally, the inhibition of gut bacteria enzymes contributing to the bacteria-derived uremic toxin production process has been suggested to offer therapeutic potentials of pharmacological intervention in uremic toxin production by gut microbiome. However, until now, only animal studies have been performed in this field. For example, Wang et al. [133] demonstrated that a structural analog of choline, 3,3-dimethyl-1-butanol (DMB) non-lethally inhibited trimethylamine formation from cultured microbes, impeded distinct microbial trimethylamine lyases reducing TMAO levels in mice fed a high-choline or L-carnitine diet which resulted in the inhibition of macrophage foam cell formation and atherosclerotic lesion development. Therefore, it seems that targeting gut microbial production of TMA with specific and non-lethal microbial inhibitors may serve as a potential therapeutic approach for the treatment of cardiometabolic diseases [133].

## 6. Conclusions

Recently, the knowledge of the metabolic potential of gut microbiome and its vital role in the pathogenesis of several chronic inflammatory diseases has widened rapidly. The gut seems highly attractive as a future target for attenuating uremia-related complications. However, there is still a need for subsequent studies focusing on the confirmation of gut microbiome pattern in kidney diseases and the analysis of associations between different types of kidney diseases and the gut microbiome. It has already been demonstrated that the presence of chronic kidney disease may be accompanied by the development of intestinal inflammation and epithelial barrier impairment leading to hastened systemic translocation of the bacterial-derived uremic toxins (e.g., p-cresyl sulphate, indoxyl sulphate, TMAO, etc.) and consequent oxidative stress injury to the kidney as well as to cardiovascular and endocrine systems. These findings have provided new therapeutic possibilities for the management of uremia, inflammation and kidney disease progression and the prevention of adverse outcomes in CKD patients. Numerous interventions aiming to restore proper gut microbiota composition and to slow the progression of kidney diseases have been examined. It seems that dietary interventions comprising prebiotics, probiotics, and synbiotics could pose a promising strategy in the management of uremic toxins in CKD. However, due to great variability of studies (in terms of the type used and the dose of fiber/prebiotic/probiotic/synbiotic) conducted so far, as well as the small number of trials’ participants, short follow-up and/or lack of dietary control make the formulation of final conclusions is impossible. It seems that more detailed research focused on the gut as a potential source of CKD-related complication may bring interesting discoveries. Despite the lack of conclusive results concerning the beneficial impact of the aforementioned compounds, a healthy dietary pattern comprising large amounts of whole fruits, grains, and vegetables should be encouraged in CKD patients due to their confirmed advantageous effect beyond uremic toxin production. However, it should be kept in mind that some fruits and vegetables contain high potassium levels and therefore, their consumption must be limited for many patients with advanced CKD or ESRD. It seems that isolated intervention involving just one type of food may not be effective in restoring proper gut microbial composition. The restoration of functionality and composition of microbiota in CKD patients requires the use of combined measures acting synergistically. Now, in the age of personalized medicine, nutritional interventions should be based on individual microbiota characteristics as well as the prevalence of comorbidities due to the fact that gut microbiota is specific to each person, and comorbidities may alter health requirements of patients. However, also these fields also require an extensive research.

## Figures and Tables

**Table 1:** Adverse impact of uremic toxins.

Design of Study	Study Group	Adverse Effect	Ref.
**ANIMAL STUDIES**			
Uremic toxins administration to subtotally nephrectomized rats	HA-treated rats vs. non-HA-treated controls, IAA-treated rats vs. non-IAA-treated controls	➢ Significantly lower GFR in uremic toxin-treated rats vs. control rats; ➢ Considerably higher β-N-acetyl-glucoseamidase excretion in uremic toxin-treated rats vs. control rats; ➢ Significantly increased glomerular sclerosis index in uremic toxin-treated rats vs. control rats; ➢ Substantial enlargement of interstitial fibrosis in IAA-treated rats. **Conclusions**: Overload of uremic toxins accelerates the loss of kidney function, glomerular sclerosis and tubulointerstitial injury in a rat model of chronic renal failure. Early intervention to remove various uremic toxins may be beneficial in delaying the onset of end-stage renal failure in patients with progressive renal disease.	[66]
Analysis of effect of indole on ECs	Germ free mouse model	➢ Decreased expression of junctional complex molecules in colonic ECs. ➢ The feces of mice contained a high amount of indole ➢ Oral administration of indole-containing capsules increased expression of both tight junction- and adherens junction-associated molecules in colonic ECs ➢ Higher resistance to dextran sodium sulfate-induced colitis. **Conclusions**: Beneficial role of indole in establishing an epithelial barrier in vivo.	[101]
Animal model study	Mouse proximal renal tubular cells (PKSV-PRs) treated with IS or PCS Half-nephrectomized B-6 mice treated with IS or PCS for 4 weeks.	➢ Significant activation of intrarenal RAAS by IS and PCS ➢ Increased renin, angiotensinogen, and angiotensin 1 (AT1) receptor expression, and decreased AT2 receptor expression in vitro and in vivo. ➢ IS and PCS significantly increased TGF-β1 expression and activated the TGF-β pathway by increasing Smad2/Smad2-P, Smad3/Smad3-P, and Smad4 expression. ➢ The expression of the EMT-associated transcription factor Snail was increased by IS and PCS treatment. ➢ IS and PCS induced the phenotype of EMT-like transition in renal tubules by increasing the expression of fibronectin and α-smooth muscle actin and decreasing the expression of E-cadherin. **Conclusions**: Activating the renal RAAS/TGF-β pathway has an important pathological role in chronic kidney injury caused by IS and PCS. IS and PCS may increase Snail expression and induce EMT-like transition.	[122]
Imaging mass spectrometry	Mouse model of CKD	➢ IS accumulated in muscle tissue of a mouse model of CKD. ➢ IS induced metabolic alterations such as upregulation of glycolysis, including pentose phosphate pathway acceleration as antioxidative stress response **Conclusions**: IS is a pathogenic factor for sarcopenia in CKD.	[125]
**CELLS CULTURE**			
Incubation of HUVEC with uremic retention solutes at concentrations found in uremic patients	HUVEC culture	➢ Inhibition of endothelial proliferation by p-cresol (in a dose-dependent manner) and indoxyl-sulfate. ➢ Decreased endothelial wound repair following incubation with p-cresol and indoxyl sulfate. ➢ Decrease in endothelial wound repair was less marked in the presence of albumin. **Conclusions:** Both p-cresol and indoxyl sulfate decrease endothelial proliferation and wound repair thus they could play a role in endothelial dysfunction observed in uremic patients.	[68]
Incubation of heparinized whole blood with p-cresol, p-cresylsulphate or their respective solvent control	Heparinized whole blood	➢ Significantly increased percentage of leucocytes displaying oxidative burst activity at baseline by p-cresylsulphate; ➢ No impact on oxidative burst activity of stimulated leucocytes ➢ P-cresol inhibited leucocytes burst activity after stimulation. **Conclusions**: P-cresylsulphate has a pro-inflammatory effect on unstimulated leucocytes. This effect could contribute to the propensity to vascular disease in the uraemic population.	[69]
Incubation of J774A.1 macrophages with IS at concentrations observed in uremic patients (1000-62.5 µM)	J774A.1 macrophages	➢ IS alone induced release of ROS via mechanisms mediated by pro- and anti-oxidant systems (LPS-induced NF-kB nuclear translocation), and alteration in intracellular calcium homeostasis (mitochondrial calcium overloading). ➢ Significant increase in NO release, iNOS and COX-2 expression induced by IS in the presence of LPS. ➢ IS pre-treatment enhanced TNF-α and IL-6 production by LPS-stimulated macrophages **Conclusions**: IS enhances the inflammatory response to LPS increasing ROS, NO, iNOS, COX-2, TNF-α, IL-6 and NF-kB levels. IS stimulates macrophage function and enhances inflammatory response associated with LPS, thus contributing to altered immune response dysfunctions observed in CKD.	[70]
Renal cortex of 5/6-nephrectomized uremic rats given indoxyl sulfate	Renal cortex of 5/6-nephrectomized uremic rats	➢ Administration of IS for five weeks significantly increased the mRNA levels of TGF-beta 1, TIMP-1 and pro-alpha 1(I) collagen ➢ Significant decline in renal function and worsening of glomerular sclerosis. ➢ Administration of IS for 2.5 weeks also increased the expression of the mRNA levels with no significant decline in the renal function. **Conclusions**: Overload of IS on remnant nephrons is involved in the increased bioactivity of TGF-beta 1 in uremic kidneys, which enhances the renal expression of TIMP-1 and type 1 collagen, leading to the progression of CRF.	[104]
Examination of the effect of IS on rat VSMC proliferation	Rat VSMC	➢ Concentration of IS needed to stimulate the proliferation of rat VSMC were compatible with that in the serum of ESRD patients (250 μM) ➢ PD98059 (a selective inhibitor of MAPK/extracellular signal-regulated kinase), inhibited the IS-induced VSMC proliferation and phosphorylation of MAPK. ➢ Probenecid (inhibitor and substrate of OAT), inhibited the IS-induced VSMC proliferation. ➢ Significantly increased mRNA of PDGF-C chain and PDGF-beta receptor by IS. **Conclusions**: IS directly stimulates rat VSMC proliferation and activates MAPK in vitro. This might be one of the mechanisms underlying the progression of atherosclerotic lesions in end-stage renal disease patients.	[105]
Analysis of direct effect of IS at uraemic concentrations on OCL differentiation and bone-resorbing activity	Well-established cellular models of monocyte/macrophage (peripheral blood mononuclear cells and the RAW 264.7 cell line).	➢ IS inhibited OCL differentiation and bone-resorbing activity in a dose-dependent manner ➢ IS induced a gradual inhibition of JNK, Akt, p38, ERK1/2 phosphorylation and AP-1 DNA-binding activity. ➢ The effects of IS on OCL differentiation and AP-1 were prevented by probenecid **Conclusions**: IS not only inhibits osteoblast function but also has an inhibitory effect on OCL function and thus could affect bone remodeling in CKD patients.	[110]
**HUMAN STUDIES**			
Cross-sectional observational study included a convenience sample of 327 participants with normal kidney function, non-dialysis CKD and end-stage kidney disease (ESKD) patients	327 participants with kidney function categorized as normal, CKD and ESKD.	➢ Significant increase in total and free IS and PCS and their free fractions across the CKD spectrum (highest levels in ESKD) ➢ Significantly greater total and free concentrations of PCS within each CKD stage than IS (all *p* < 0.01). ➢ Correlation between IS and PCS, free and total, and BAR-GTN (ranging from *r* = −0.33 to −0.44) and cIMT (*r* = 0.19 to 0.21) ➢ Independent association between all toxins and the presence of cardiovascular disease (all *p* < 0.02). **Conclusions**: More advanced stages of CKD are associated with progressive increases in total and free serum IS and PCS, as well as increases in their free fractions. Total and free serum IS and PCS were independently associated with structural and functional markers of CAD.	[72]
Prospective observational study	268 patients with different stages of CKD	➢ Relationship between high-serum PCS levels and renal progression and all-cause mortality independent of age, gender, diabetes status, albumin levels, serum IS, serum creatinine, Ca × P product, intact parathyroid hormone, hemoglobin or high-sensitivity C-reactive protein level. ➢ Serum IS was only associated with renal progression; **Conclusions**: Serum IS and PCS levels may help in predicting the risk of renal progression in patients having different stages of CKD.	[128]
Observational study	139 patients with different stages of CKD	➢ Inverse relationship between baseline total and free PCS with renal function a ➢ Significant correlation between PCS and vascular calcification. ➢ Free (but not total) PCS was a predictor of overall and CV death. ➢ Higher free PCS levels (>0.051 mg/100 mL; median) were associated with mortality independently of age, vascular calcification, anemia and inflammation. **Conclusions**: Serum levels of free and total PCS were elevated in later CKD stages. Free PCS seems to be a predictor of survival in CKD.	[69]
Observational study	103 stable CKD patients (stage 3–5 and hemodialysis (HD) patients)	➢ Higher serum IS, PCS in patients with advanced CKD ➢ Significant correlation between IS and PCS and serum creatinine after multivariate regression analysis (*B* = 3.59, *p* < 0.01; *B* = 0.93, *p* = 0.04, respectively). ➢ Positive correlation between IS and PCS level (*r* = 0.61, *p* < 0.01). **Conclusions**: IS and PCS level increased gradually while renal function declined and reached the peak at the stage of HD.	[78]
Observational study	120 patients with CKD	➢ Significantly higher mortality and cardiovascular events in the higher IAA group (IAA > 3.73 µM) vs. lower IAA group (IAA < 3.73 µM). ➢ Serum IAA—a significant predictor of mortality and cardiovascular events ➢ Positive correlation between IAA levels and markers of inflammation (CRP) and oxidative stress (malondialdehyde) ➢ IAA increased production of endothelial ROS. **Conclusions**: Serum IAA may be an independent predictor of mortality and cardiovascular events in patients with CKD.	[77]
Observational study	100 stable and eligible HD patients	➢ Total and free PCS correlated with right and left ABI and PWV (*p* < 0.01), and total IS was associated with right and left ABI (*p* < 0.01) ➢ Dialysis length and total PCS was associated with to arteriovenous -shunt failure event (HR: 1.14, *p* = 0.01, and HR: 1.04, *p* = 0.04, respectively). ➢ Total and free PCS and IS were positively linked to numbers of PTA and thrombectomy. ➢ Total PCS was significantly associated with vascular access failure event (log rank *p* = 0.02). **Conclusions**: Serum levels of PCS and IS were associated with PAD and total PCS could be a valuable determinant of access viability other than traditional or nontraditional risk factors in HD patients.	[107]
Cross-sectional observational cohort study	Participants with stage 3–4 CKD	➢ Independent association between serum free and total IS were with serum IL-6, TNF-α and IFN-γ, ➢ Independent association between serum free and total PCS and serum IL-6 and PWV. **Conclusions**: IS and PCS were associated with elevated levels of selected inflammatory markers and an antioxidant in CKD patients. PCS was also associated with increased arterial stiffness. Inflammation and oxidative stress may contribute to the nephro- and cardiovascular toxicities of IS and PCS. I	[129]
Observational study	4007 patients undergoing elective coronary angiography	➢ Markedly suppressed plasma levels of TMAO after the administration of antibiotics ➢ Increased plasma levels of TMAO were associated with an increased risk of a major adverse cardiovascular event (HR for highest vs. lowest TMAO quartile, 2.54; 95% Cl, 1.96 to 3.28; *p* < 0.001). **Conclusions**: The production of TMAO from dietary phosphatidylcholine is dependent on metabolism by the intestinal microbiota. Increased TMAO levels are associated with an increased risk of incident major adverse cardiovascular event	[135]
Observational study	CKD cohort (*n* = 104) CKD cohort (*n* = 220) undergoing coronary angiography	➢ Strong inverse association between serum TMAO concentrations and eGFR (*r*(2) = 0.31, *p* < 0.001). ➢ TMAO concentrations were markedly higher in patients receiving dialysis ➢ Renal transplantation resulted in substantial reductions in TMAO concentrations ➢ TMAO concentration was an independent predictor for coronary atherosclerosis burden (*p* = 0.02) and predicted long-term mortality independent of traditional cardiac risk factors (HR, 1.26 per 10 μM increment in TMAO concentration; 95% CI, 1.13 to 1.40; *p* < 0.001). **Conclusions**: Serum TMAO concentrations substantially increase with decrements in kidney function, and this effect is reversed by renal transplantation. Increased TMAO concentrations correlate with coronary atherosclerosis burden and may associate with long-term mortality in patients with CKD undergoing coronary angiography.	[139]
Longitudinal study	CKD patients ranging from mild-moderate to ESRD	➢ GFR was the dominant variable affecting TMAO (β = −0.41; *p* < 0.001), choline (β = −0.38; *p* < 0.001), and betaine (β = 0.45; *p* < 0.001) levels. ➢ Dialysis treatment did not affect TMAO, ➢ Renal transplantation reduced levels of TMAO to that of controls (*p* < 0.001). ➢ In CKD 3–5, TMAO levels were associated with IL-6 (Rho = 0.42; *p* < 0.0001), fibrinogen (Rho = 0.43; *p* < 0.0001) and hsCRP (Rho = 0.17; *p* = 0.022). ➢ Higher TMAO levels were associated with an increased risk for all-cause mortality that remained significant after multivariate adjustment (HR 4.32, 95% CI 1.32–14.2; *p* = 0.016). **Conclusion**: Elevated TMAO levels are strongly associated with degree of renal function in CKD and normalize after renal transplantation. TMAO levels correlates with increased systemic inflammation and is an independent predictor of mortality in CKD 3–5 patients.	[140]

ABI—ankle brachial index; BAR-GTN—brachial artery response to glyceryl trinitrate; CAD—cardiovascular disease; CAPD—continuous ambulatory peritoneal dialysis; EMT—tubular epithelial-to-mesenchymal transition; cIMT—carotid intima-media thickness; Cl—confidence interval; COX-2—cycloxygenase-2; CRF—chronic renal failure; CRP—C-reactive protein; ESKD—end-stage kidney disease; HD—hemodialysis; HR—hazard ratio; HUVEC—human umbilical vein endothelial cells; IAA—indole acetic acid; IL-6—interleukin-6; iNOS—inducible nitric oxide synthase; IS—indoxyl sulphate; MAPK—mitogen-activated protein kinase; NO—nitric oxide; LPS—lipopolysaccharide; OCL—osteoclast; OR—odds ratio; PCS—p-cresyl sulphate; PDGF—platelet-derived growth factor; PTA—percutaneous transluminal angioplasty; PWV—pulse wave velocity; RAAS—renal renin-angiotensin-aldosterone system; ROS—reactive oxygen species; RTR—renal transplant recipients; TGF-β1—transforming growth factor-β1, TIMP—tissue inhibitor of matrix proteinase; TMAO—trimethylamine-N-oxide; TNF-α—tumor necrosis factor-α; VMSC—vascular smooth muscle cell (VSMC).

## Data Availability

No additional data is available.

## Abbreviations

ABI	ankle brachial index
BAR-GTN	brachial artery response to glyceryl trinitrate
CAD	cardiovascular disease
CAPD	continuous ambulatory peritoneal dialysis
cIMT	carotid intima-media thickness
CKD	chronic kidney disease
Cl	confidence interval
COX-2	cycloxygenase-2
CRF	chronic renal failure
CRP	C-reactive protein
EMT	tubular epithelial-to-mesenchymal transition
ESKD	end-stage kidney disease
ESRD	end-stage renal disease
FGF 23	fibroblast growth factor 23
GBM	glomerular basement membrane
HD	hemodialysis
HR	hazard ratio
HUVEC	human umbilical vein endothelial cells
IAA	indole acetic acid
IL-6	interleukin-6
iNOS	inducible nitric oxide synthase
IS	indoxyl sulphate
IRI	ischemia reperfusion injury
LPS	lipopolysaccharide
MAPK	mitogen-activated protein kinase
NO	nitric oxide
OAT1, OAT3	organic anion transporters
OCL	osteoclast
OR	odds ratio
OTUs	operational taxonomic units
PAG	Phenylacetylglutamine
PBUT	protein-bound uremic toxins
pCG	p-cresyl glucuronide
PCS	p-cresyl sulphate
PDGF	platelet-derived growth factor
PTA	percutaneous transluminal angioplasty
PTH	parathormon
PWV	pulse wave velocity
SDGT	Sodium-dependent glucose cotransporters
RAAS	renal renin-angiotensin-aldosterone system
ROS	reactive oxygen species
RTR	renal transplant recipients
SCFA	Short chain fatty acids
TGF-β1	transforming growth factor β1
TIMP	tissue inhibitor of matrix proteinase
TLRs	toll-like receptors
TMAO	trimethylamine N-oxide
TNF-α	tumor necrosis factor-α
VMSC	vascular smooth muscle cell
